# Complete Genomic Sequence of the Thermophilic Hydrogen-Oxidizing Methanogen Methanothermobacter tenebrarum Strain RMAS^T^

**DOI:** 10.1128/mra.00355-22

**Published:** 2022-07-11

**Authors:** Kohei Nakamura, Kenshiro Oshima, Masahira Hattori, Yoichi Kamagata, Kazuhiro Takamizawa

**Affiliations:** a Faculty of Applied Biological Sciences, Gifu University, Gifu, Japan; b Center for Omics and Bioinformatics, Graduate School of Frontier Sciences, The University of Tokyo, Kashiwa, Chiba, Japan; c Bioproduction Research Institute, National Institute of Advanced Industrial Science and Technology, Tsukuba, Ibaraki, Japan; Portland State University

## Abstract

Methanothermobacter tenebrarum strain RMAS^T^ has a complete genomic length of 1,472,762 bp, a GC content of 42.1%, 1,599 coding DNA sequences (CDSs), 1 CRISPR array, 3 rRNAs, and 38 tRNAs.

## ANNOUNCEMENT

Methanothermobacter tenebrarum strain RMAS^T^ is a thermophilic and hydrogenotrophic methanogen that was isolated from the gas-associated production water of a gas-producing well in a natural gas field in Japan (1). Uncultured methanogens closely related to *M. tenebrarum* RMAS^T^ are often reported as the predominant methanogens in methanogenic digesters and subsurface environments (2, 3). The genomic information of *M. tenebrarum* RMAS^T^ could help distinguish it from related strains, such as Methanothermobacter thermautotrophicus strain ΔH^T^. Therefore, we report the complete genomic sequence of strain RMAS^T^.

Strain RMAS^T^ was cultured from stock maintained in the laboratory (1). Cells were recovered by centrifugation and used for DNA extraction. Genomic DNA was extracted using a conventional method based on phenol-chloroform extraction (4). The complete genome sequence for RMAS^T^ was determined using a whole-genome shotgun method with Sanger sequencing (5). The extracted DNA (5 μg) was sheared mechanically using a HydroShear device (GeneMachines) for construction of DNA libraries with short (1 to 3 kb) and long (>5 kb) inserts. The sheared DNA was ligated into the pUC18 plasmid and transformed into Escherichia coli DH5α. Colony-direct PCR was performed to prepare a DNA template, and the BigDye Terminator cycle sequencing kit and ABI3730xl DNA analyzer (Applied Biosystems) were used to sequence the genome. Sequencing yielded 13,720 and 6,516 double-ended sequence reads (13-fold coverage) with median lengths of 1,076 and 1,082 bp for the short- and long-insert templates, respectively.

Default parameters were used for all software unless otherwise specified. The read data were assembled and edited and the sequence finished using the Phred/Phrap/Consed v.030415 package (6). Sequence data with ≥200 bases and a Q score of >20 were used for the assembly. PCR products amplified with appropriate primers were sequenced to resolve the gaps. Assembling the sequences covering the gaps and other data yielded two gap-closed consensus sequences with different lengths (1,472,762 and 22,428 bp). In the final assembly, 99.9998% of the sequence had a consensus Q score of ≥40.

The assembled sequences were annotated using DFAST (7) and Prokka v.1.14.6 (8). We have concluded that the smaller and larger sequences were derived from plasmid DNA and genomic DNA, respectively, since their sequence statistics showed an apparent difference in GC content between them. The replication origin of the genome was predicted using an optional function implemented in DFAST. The RMAS^T^ genome is a circular sequence with a length of 1,472,762 bp, a GC content of 42.1%, 1,599 coding DNA sequences (CDSs), 3 rRNAs, 38 tRNAs, and 1 CRISPR. The plasmid is a sequence 22,428 bp long, with a GC content of 38.4% and 36 CDSs.

Average nucleotide identity (ANI) analysis using FastANI (9) showed no apparent ANI values between strain RMAS^T^ and the genus *Methanothermobacter* with validly published names and complete genomes. Closely related RMAS^T^ strains were found as metagenome-assembled genomes (GenBank accession numbers GCA_012840175.1 [ANI, 99.88%] and GCA_001507955.1 [ANI, 87.59%]) that were retrieved from an anaerobic digester and an oil reservoir, respectively (10, 11).

Strain RMAS^T^ has no genes related to acetyl coenzyme A (acetyl-CoA) decarbonylase/synthase, necessary for CO_2_ fixation for autotrophic hydrogenotrophic methanogens. The strain RMAS^T^ genome uniquely contains a single set of Mcr genes (MTTB_02540 to MTTB_02580), while other strains of the genus *Methanothermobacter* with complete genomes carry two Mcr gene copies.

### Data availability.

The complete genome sequence of Methanothermobacter tenebrarum strain RMAS^T^ and its plasmid DNA sequence have been deposited at DDBJ under the accession numbers AP025698 and AP025699, respectively. The versions described in this paper are versions AP025698.1 and AP025699.1, respectively. The raw sequence reads were deposited in the DDBJ Sequence Read Archive under accession number DRA013751, with BioSample accession number SAMD00451045 and BioProject accession number PRJDB13305.
